# GENOME-WIDE ASSOCIATION STUDY OF EXTREME HUMAN LONGEVITY DISCOVERS UNCOMMON LONGEVITY VARIANTS

**DOI:** 10.1093/geroni/igz038.759

**Published:** 2019-11-08

**Authors:** Anastasia Gurinovich, Anastasia Gurinovich, Zeyuan Song, Stacy L Andersen, Thomas T Perls, Paola Sebastiani

**Affiliations:** 1 Boston University, Boston, Massachusetts, United States; 2 Bioinformatics Program, Boston University, Boston, Massachusetts, United States; 3 Department of Biostatistics, Boston University School of Public Health, Boston, Massachusetts, United States; 4 Department of Medicine, Boston University School of Medicine, Boston, Massachusetts, United States

## Abstract

The strong heritability of extreme human longevity supports the hypothesis that this is a genetically-regulated trait. However, association studies focused on common genetic variants have discovered a limited number of longevity-associated genes. We conducted a genome-wide association study of 4,216 individuals including 1317 centenarians from the New England Centenarian Study (median age = 104 years) using >9M genetic variants imputed to the HRC panel of ~65,000 haplotypes. The set included approximately 5M uncommon variants. The associations were tested using a mixed effect logistic regression model with genotype-based kinship covariance of the random effects to adjust for cryptic relations using the package GENESIS. The analysis discovered 45 genome-wide significant SNPs (p< 5E-08) including 8 new loci in chromosomes 3, 6, 7, 9, 10, 14 and 15 in addition to the APOE locus. The list includes new pQTLs in serum that suggest a new biological mechanism involved in extreme human longevity.

